# Details of the 1998 Watt Balance Experiment Determining the Planck Constant

**DOI:** 10.6028/jres.110.003

**Published:** 2005-02-01

**Authors:** Richard Steiner, David Newell, Edwin Williams

**Affiliations:** National Institute of Standards and Technology, Gaithersburg MD 20899-8112 USA

**Keywords:** absolute watt measurement, electronic kilogram, electronic watt determination, fundamental physical constants, Planck constant, SI power measurement, SI watt unit, watt balance

## Abstract

The National Institute of Standards and Technology (NIST) watt balance experiment completed a determination of Planck constant in 1998 with a relative standard uncertainty of 87 × 10^−9^ (*k* = 1), concurrently with an upper limit on the drift rate of the SI kilogram mass standard. A number of other fundamental physical constants with uncertainties dominated by this result are also calculated. This paper focuses on the details of the balance apparatus, the measurement and control procedures, and the reference calibrations. The alignment procedures are also described, as is a novel mutual inductance measurement procedure. The analysis summary discusses the data noise sources and estimates for the Type B uncertainty contributions to the uncertainty budget. Much of this detail, some historical progression, and a few recent findings have not been included in previous papers reporting the results of this experiment.

## 1. Introduction

The watt balance is an experiment with historical roots in the absolute-ampere current balance built at the National Bureau of Standards (NBS) [1]. This relied upon measuring forces between a solenoid and an induction coil where the magnetic flux was calculated from dimensional measurements. The development of quantum voltage and resistance standards in the intervening years allowed the experiment to be redesigned. Now, mechanical power is measured in units of mass, length, and time, then compared to electrical power as measured in units of voltage and resistance. Because of the quantum electrical standards involved, the ratio of the two power values results in a direct measurement of *h*, the Planck constant. In September 1998, the National Institute of Standards and Technology completed a new evaluation of the watt and reported the corresponding value for *h*. This was the cumulative work of 10 years of improvements over the last reported value. Many research articles recorded this progress, so the goal of this paper is to combine the previous reports into a coherent whole, adding experiment details bypassed in the shorter briefs. The organization of this paper is as follows. Section 1/1.1 summarizes the theory of how the watt ratio result relates to the calculation of the Planck constant. Section 2 describes the experimental apparatus along with the procedures that were used in calibrating or operating the equipment. Section 3 reviews the calibration reference measurements needed to keep the system in a sufficiently characterized state and the resulting Type B error analysis. The watt data acquisition procedures are discussed in Sec. 4 and the signal analysis methods are discussed in Sec. 5. A brief epilog mentions some major changes made in the latest version of the experiment.

### 1.1 Watt Data to Planck Constant Theory

**Fig. 1:**
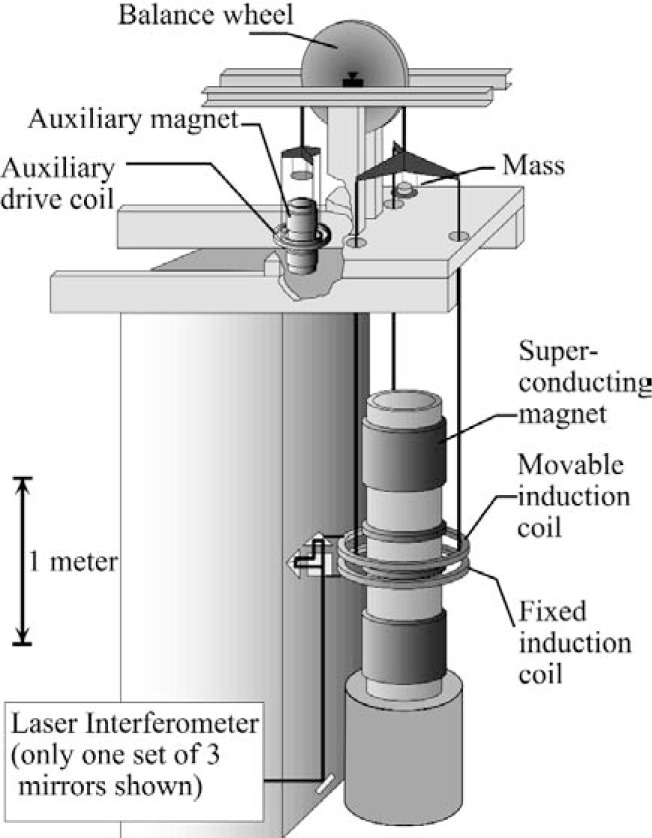
A schematic diagram of the watt balance experiment.

For decades, current balance experiments were used to maintain electrical standards. In 1976, Kibble [2] realized that the same apparatus could compare mechanically generated power to electrical power, without the need for calculating the field, by adopting a two mode measurement scheme. The apparatus schematic, showing the 1998 NIST configuration, is in Fig. 1. It is essentially a force balance using a pulley with a knife edge pivot, capable of acting as either an electric generator or electric motor. In the first of the two measurement modes, the inductive coil is moved vertically at some velocity to generate a dc voltage. Timed measurements of the generated voltage, *U*, between travel positions to get the average velocity, *v*, provide a ratio *U*/*v* that is dependent on the magnetic flux, *Φ*,

$$U=-\frac{d\Phi }{dt}=-\frac{dz}{dt}\frac{\partial \Phi }{\partial z}=-v_{z}\frac{\partial \Phi }{\partial z}.$$ (1)

In the second mode, a current, *I*, is passed through the coil to create an electrical force that statically balances the mechanical force on a kilogram mass in Earth’s gravity, *mg*. Measuring the current provides a second ratio *mg*/*I*, also dependent on the flux,

$$F_{z}=mg=-I\frac{\partial \Phi }{\partial z}.$$ (2)

The flux within the induction coil is the integral of the flux density ***B*** over the area segment d***a***, ***Φ*** = ∫***B*** · d***a***. Designing the solenoid for a flux density spatial dependence of 1/*r* means that the flux gradient is virtually constant for any shape of the induction coil (more on this later). The ratio of the measured quotients of *U*/*v* and *F*/*I* both depend on the flux gradient, thus eliminating the dependence on magnetic field. Reordering the variables, we obtain a ratio of mechanical power to electrical power, which is the central watt balance equation,

$$\frac{mg/I}{U/v}=\frac{mgv}{UI}.$$ (3)

Since there is no motion during force measurements, no energy is lost to friction. With the induction coil open (digital voltmeter, or DVM, very high impedance), there is virtually no current flow, and thus, no significant term for energy lost to resistive heating. This has been referred to as a virtual power measurement. Theory understands that these power calculations are equivalent, with units in watts, W, in the International System of Units, (SI). However in this instance, there can be a measured difference in these power determinations, due to differing units of measure. To explain, consider two values and their units, *L*_1_ = {1} inch and *L*_2_ = {2.54} cm, using curly brackets to separate the numerical values from the units of measure. *L*_1_/*L*_2_ are the same lengths but with different units, so their ratio equals 1, while the ratio 1/2.54 inversely reflects the units’ ratio of inch/cm. With this convention of values and units, power formulas can be written as,

(F·v)z={mgvz}W=UI={U2Z}W. (4)

From Ohm’s law, power is dimensionally equal to

$$W=V^{2}/\Omega ,$$ (5a)

with units of ohms, Ω, and volts, V. The realized electrical references, however, are not base units in SI, but by convention are based on representational units. Thus the electrical units, as adopted in 1990,

$$W_{90}={V_{90}}^{2}/\Omega_{90},$$ (5b)

are only a practical realization of the SI units. These electrical references have highly reproducible representations, based on the discoveries in 1962 of the Josephson effect [3] and in 1980 of the quantum Hall effect [4]. Voltage is proportional to frequency, *f*, via the Josephson effect with a constant, *K*_J_ = 2*e*/*h*, where *e* is elementary charge. Although well understood theoretically, [the third part of Eq. (6)] the value for *K*_J_ is not known perfectly, so a practical value *K*_J−90_ = 483 597.9 MHz/V was adopted in 1990. To distinguish these measured voltage units, the unit symbol V_90_ is used,

U=nfh2e={nfKJ}V={nfKJ−90}V90, (6)

where *n* is a quantum number. Similarly, resistance, *Z*, is proportional to the von Klitzing constant, *R*_K_ = *h*/*e*^2^, via the quantum Hall effect. As with voltage, *R*_K−90_ = 25 812.807 Ω is an adopted value, and

Z=(hie2)={RKi}Ω={RK−90i}Ω90, (7)

where *i* is a quantum number. It’s useful to note from these theories that the constants in *K*_J_ and *R*_K_ can be combined and reduced to

KJ2RK=(2e/h)2(h/e2)=4h. (8)

Using the unit power equivalencies of Eq. (4), a power measurement can be written using the theoretical constants and SI units,

mgv=U2Z={nfKJ}2{iRK}W. (9a)

This is equal to the same equation in representational units,

U2Z={U2Z}90W90={nfKJ−90}2{iRK−90}W90. (9b)

Take the ratio of Eqs. (9a) and (9b) and we are back to Eq. (3), where the ratio of power measurements can be written in terms of the appropriate measurement units,

(F·v)z(UI)={mgv}W{U2Z}90W90={nfKJ}2{iRK}W{nfKJ−90}2{iRK−90}W90≡1in SI. (10)

This is the basic watt balance equation. The units cancel, as do the frequency and quantum number variables (they are used only in determining the reference standards), and only the actual measured values of the experiment become useful. An interesting note in this equation is that the values for *K*_J−90_ and *R*_K−90_ do not have to be known at all. As long as they are used consistently for all electrical measurements, the difference between the assigned constants and the “perfect” SI value for those constants simply gives a watt ratio differing from 1. Finally, in combination with Eq. (8), the watt balance measurement results leads to a determination of the Plank constant,

({mgv}SI/{U2Z}90)/{KJ−902RK−90}=h4. (11)

However, a significant outgrowth of the watt balance now becomes apparent. The references for the equation variables, *g*, *v*, *U*, *I*, are length, time, voltage, and resistance; all quantum based standards. Mass *m* is relative to an artifact standard, the SI International Prototype Kilogram (IPK). Therefore, over time, if the watt measurement implies that the aforementioned watt ratio drifts consistently (Planck constant changes?), then the implication must be that the artifact mass standard must be changing. This is the idea that is now driving further development of the watt balance into an electronic method for monitoring IPK [5].

In our previously reported results on the determination of Planck constant, *h* [6,7], the uncertainty was estimated at 8.7 × 10^−8^. (All uncertainties are relative standard uncertainties with *k* = 1, unless otherwise specified.) This is a factor of 2 smaller than reported by Kibble et. al. [8]. The close agreement with his result of 1988 gives an upper limit for the drift of the SI kilogram of (1 ± 2) × 10^−8^ per year. The next sections describe this experiment apparatus and its procedures, along with more detail of the various components of the uncertainty budget.

## 2. Apparatus Descriptions

The watt balance physical construction is illustrated schematically in Fig. 1. At it core it is a mass balance. Suspended below the main mass pan is an induction coil, residing in the magnetic field of a superconducting solenoid. The force of gravity on the test mass can be balanced by electromagnetic force produced in the coil. The watt balance experiment resides in an insulated, temperature controlled room within the Nonmagnetic Facility on the NIST grounds, located away from the main research buildings. To minimize air currents, the room has no forced air ventilation. There are two smaller enclosures, which will be referred to as the upper box and the lower box. The upper box houses the balance mechanism. The walls are heavily insulated for additional temperature stability and further reduction of air currents. The lower box encloses the induction coils, also to reduce air currents.

### 2.1 Balance and Induction Coil

#### 2.1.1 Main Mass Side

The NIST watt balance design differs from a normal precision mass balance by using a pulley wheel mechanism instead of a lever arm, and a knife-edge pivot instead of a flexure. The pulley, or balance wheel, avoids the horizontal displacement cosine error that is a function of rotating a lever arm. The wheel is about 61 cm in diameter, made of aluminum with a stainless steel band welded to the rim. The steel is polished to provide smooth movement and resist wear from the support bands. Since there is ±10° of rotation of the wheel involved in the velocity mode, using a knife-edge avoids the severe bending stress that would result on a flexure. The knife-edge rests on a rectangular piece of polished, optically flat boron carbide. Different materials were tried as knife edges, including quartz and sapphire, but these tended to deteriorate too rapidly. TaW alloy was used during the later stages of the experiment and displayed little wear after about 1.5 years of use. The wheel’s axis lies along an east-west direction, which defines an *x*-axis dimension. The mass pan and induction coil are suspended from a tri-arm spider support structure, which is in turn fastened to the balance wheel about 45° from the top with a flat band of wires.

The band consists of 50 PtW-alloy wires, each wire nominally 120 µm in diameter. A non-overlapping, multi-wire construction averages out the thickness variations in the wires. This helps reduce moment arm variations as the band turns off the balance wheel, which smoothes out rapid velocity changes. Each band is made by winding the wire under constant tension between two screw shafts, which are 1.5 cm in diameter, 317 µm pitch separation, and 41 cm apart. The 50 strands are soldered onto a brass tongue of 0.2 mm shim stock. To “untwist” the band, i.e., reduce the torque-inducing tendency of the band to coil from natural twists in the wire, the band is heat treated with a propane torch. A natural twist in the unloaded band of up to 10° was acceptable.

Another integral component of the main mass side of the balance is the induction coil system, one movable and one fixed. Both have dimensions 33.6 cm ID, 37.2 cm OD, and 2.5 cm thick. The moveable coil is located 3 m below the mass pan, centered and in air, around a Dewar containing a superconducting magnet. Each coil was wired as three concentric coils of 785 turns each, wet laid with epoxy while winding onto a Teflon form. The Teflon was later removed and aluminum foil was epoxied onto the surface of the coil to reduce static charges and forces. The three-coil aspect allowed different wiring configuration choices: any one of the three single coils, any two in series opposition, or all three in series. An identical coil was in a fixed position about 7 cm below the moving coil. During voltage measurements, the two induction coils are wired in series opposition to cancel background ac. This also rejects common mode motion of the coils relative to the magnetic flux, and thus the fixed coil could be used as *z*-axis reference for the displacement of the moving coil. For current measurements in the force mode, the moving coil was wired alone in series with a reference standard 100 Ω resistor.

#### 2.1.2 Measuring Position—Laser Interferometer Systems

**Fig. 2:**
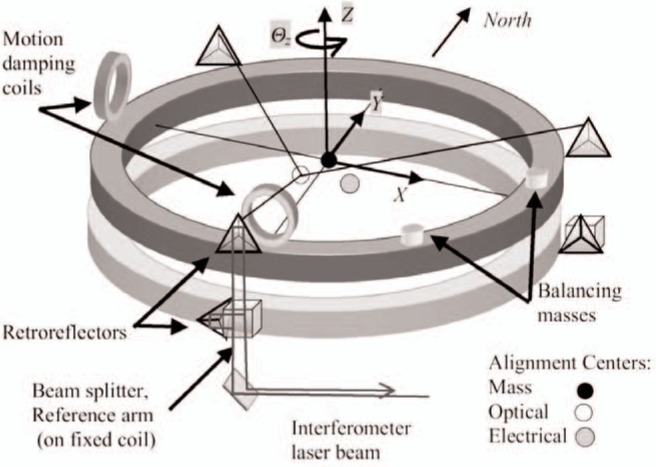
The moving induction coil (dark) and fixed coil (light), with an overlay of the coordinate axes. Some components built onto the moving coil are also shown, as are the different centers as referenced in the alignment section.

Several laser optic systems are needed to determine the position of the induction coil along various axes. One laser interferometer is located axially, with the optics in the upper box. A retroreflector is supported from the hanging spider-assembly, directly beneath the central suspension point. Since the tension on the band and the optics support structure is always constant, this interferometer determines the balance *z*-position (the wheel angle) and also provides the balance servo-feedback signal. In the earlier watt experiment apparatus, this axial interferometer on the coil’s support system was also used for velocity determination. This posed a severe problem for synchronous measurement of the coil’s position and the generated voltage. Vibrations in the 3 m support rods meant that the induction coil experienced additional oscillatory motion. This motion in turn would be visible, in part, at the axial interferometer, but with a phase error. To solve this problem, a triple-laser interferometer system was installed onto the induction coil. A single laser beam is split three ways into interferometers located 120° apart, at just outside the coil’s radius. The retroreflectors are made of three front surfaced mirrors forming a hollow corner cube. Acting as the motion reference point, a reference arm about 1 cm long on the same platform as the polarizing beam splitters, was connected to the fixed coil. Figure 2 illustrates the positions of these corner cubes on the coil.

A third laser system registers the lateral and rotational position of the induction coil. It was originally used to damp any swinging of the coil. However, it became essential for maintaining the rotation angle of the coil and in checking some of the alignments. This *X*-*Y*-*θ_z_* monitoring system consists of two photo sensors with two-dimensional position sensitivity measuring the laser beam displacement as reflected from corner cubes at 90° separation on the coil. Signals are recorded as east-west and north-south difference voltages, along with the sensor sum voltage to compensate for laser intensity variations. The four difference signals are mathematically combined to record motion in the *Y*-axis or north-south direction, *X*-axis or east-west direction, and rotation *θ_z_* around the *Z*-axis. A third photo sensor detects laser motion as reflected off a flat mirror to register tilting of the coil around either *X*- or *Y*-axis.

This detection system was originally envisioned solely as providing the feedback signal to a motion damping system. During velocity passes and especially mass placement, the induction coil experiences off-axis forces, e.g., air currents or torque from an imperfectly centered mass, and it starts to swing in a pendulum motion with 4 s period. Horizontal forces to damp this motion are provided with two auxiliary electromagnetic coils, located on the moving induction coil at the west and south positions with their areas at right angles to the radial magnetic field. With feedback from the monitoring sensors, this system damps out the coil’s swinging motion independently in each *X*–*Y* direction. These coils are activated only at the beginning of each data pass. After swinging is reduced to a negligible amount, the auxiliary coils are disconnected to prevent a closed circuit inductor, which would add torque or drag on the coil. The coil rotation *θ_z_* results from the residual curl in the wires of the support bands, which cause a torque on the coil as the band winds up and down the wheel. To keep the coil’s interferometer laser beams centered on their detectors, a constant *θ_z_* must be continuously maintained. This is done via an electrostatic rotation control system. Three glass plates are vertically aligned on each of the three support rods. Each is coated with gold film on both sides and electrically grounded. Electrodes on both sides of each plate are supplied with a variable 2000 V potential to create a force tangential to the rotation. The equilateral locations reduce any horizontal force on the coil. Electrostatic forces on the vertically aligned gold plates contribute no *Z*-axis forces. Coil tilt is not damped, but with a lower Q and a higher fundamental frequency, the tilts self-damp within a few tens of seconds.

#### 2.1.3 Counter Mass Side

The balance counter mass is made of brass, aluminum, and plastic sections combined to match the density of the main induction coil. This minimizes buoyancy drift with air pressure changes. To provide a small drive force for velocity control, two small inductive coils, spatially separated but planar and wired in series opposition, are centered around a set of permanent magnets configured to form a radial mini-field. The drive current is distributed between the coils to minimize any remnant field at the position of the main induction coils. The counter mass side also has a mass pan, as a tare mass of 0.5 kg is added to this side during force balance measurements. The entire balance assembly is standing on a limestone block, housed in a two story, thermally insulated room. The bulk of the upper box is cantilevered with wooden beam supports attached to the side of the pier. These support structures and enclosures are as massive as possible in an effort to stabilize the alignments while reducing vibration, temperature drift, and air current effects.

### 2.2 Superconducting Solenoid

Although the magnetic field dependence drops out of the watt balance equation to first order, there are desired characteristics for the magnetic field. First, the objective was to design a magnet solenoid and induction coil system to generate 1 V in the coil moving outside the Dewar. A second desire was a field amplitude as stable as possible over several hours of measurement time. Third, since the alignment of the magnetic field vector needs to be perpendicular to the earth’s gravity vector, measuring the magnetic field and tilting the solenoid is necessary. As a final consideration, Olsen [9] realized that with an induction coil’s coupling to the field depends on the coil’s radius *r*, then a magnetic field, radial and varying as 1/*r*, would cancel to first order any dimensional variations in the induction coil from temperature changes.

**Fig. 3:**
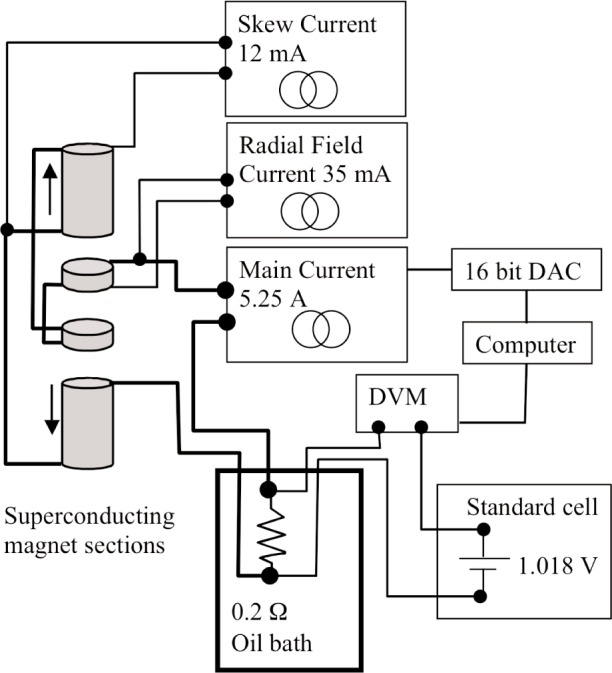
Constant current control system for the superconducting magnet.

**Fig. 4:**
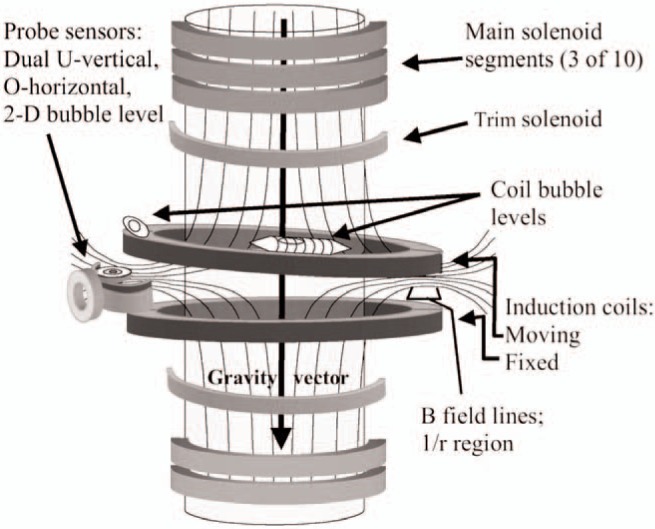
Schematic showing inductor coils, several superconducting solenoid segments, and magnetic field lines. The small sensor probe coils, moved around the solenoid, are used to set magnetic field perpendicular to acceleration vector of gravity. The tilted moving coil illustrates a typical misalignment.

Our design objectives are met by generating a field with two large superconducting solenoids wired in series opposition (Figs. 1, 3, 4). Construction requirements are eased with each solenoid consisting of 10 separate segments. All segments are bolted into position with aluminum rods. Steel tubes connect to three of the rods to provide a hanging support from the Dewar’s top flange. Combined, there are 200 000 turns for an inductance of 5800 H. The magnetic flux density at the 35 cm average radius of the induction coil for 5.25 A current is 0.1 T. Each main solenoid section has taps for independent current-compensation so that minor construction asymmetry between the two can be eliminated. A second pair of trim solenoids, located between the opposing main solenoids, are added to the design to add a second computational solution corresponding to 1/*r* dependence within the radius of the induction coil. Thus, the field is not strictly 1/*r* across the entire coil, but the currents can be adjusted to give an effective 1/*r* dependence. This is described later.

In the calculations of Sec. I, the induction coil’s coupling to the magnetic flux density of the solenoid field is considered the same with or without current in the induction coil. This is accomplished with a constant solenoid current, as opposed to the persistent current mode of the superconductor, which otherwise maintains a constant flux within the solenoid. Maintaining the current to the desired few parts in 10^8^ over minutes was achieved with an active feedback, current controller referenced to a constant voltage (Fig. 3). A 0.2 Ω resistor (with a shunting trim resistor) in the oil bath is temperature controlled to 25 °C, as is the saturated standard cell in its custom air box. The main current of 5.25 A creates a 1.018 V drop across this resistor, the difference of which from the cell voltage is measured by a DVM. A programmable current source is adjusted with high resolution by a six-digit digital/analog converter to maintain ac peak-to-peak recorded noise in a 1 Hz bandwidth to about 100 parts in 10^9^. There are no reversing measurements, so the field stability is subject to unmeasured long term drift rates or short term variations of the standard cell, DVM offset and gain (minimized by trimming the resistor to obtain near null voltage) and the temperature variations of the oil bath. The field varied typically within 1 µA/A per day.

Secondary trim current sources are used to optimize the magnetic field and are adjusted periodically at alignment tests. *Z*-axis symmetry (skew) is effected with 12 mA added to one main solenoid. The magnet was designed to have two nodes of 1/*r* dependence about 2 cm apart and within the induction coil’s thickness. The position and separation of these nodes can be adjusted by adding current (radial field trim) to both the trim solenoids. A regular procedure was developed to optimize the radial field trim, employing the separately connectable, three coils-in-series nature of the induction coil. Servo-control to the center *z* position is accomplished with only the center of the three. Meanwhile, a 20 mA test current is injected through the inner and outer coils, specially switched into series opposition. The radial field trim current is adjusted to null out the combined forces of these two coils, thus canceling the coils’ *r* dependence with the field’s 1/*r*. Extrapolating between measurements at differing trim current settings achieves a sensitivity to about 10 µA out of the approximately 35 mA. A magnetic field modeling program, adapted from the original design calculations for the superconducting solenoid [10], revealed that the non-1/*r* deviation across the induction coil’s dimensions is about 600 parts in 10^6^. Both these constant current sources have low noise and typical drift of less than 50 pA per day. Watt values obtained with these trim currents on or off were the same, so they are not considered an error source; the advantage is more for reducing the short term noise.

## 3. Preliminary Experiment Setup, Environment Control, Noise Sources

This section is divided into three segments. Part one concerns the various alignments that must be performed to begin a measurement. Part two addresses the reference calibrations that are needed. Part three describes the testing to determine the effects of various noise sources within the system.

### 3.1 Alignments

Although the reference standards or analysis techniques can be adjusted and applied to recorded data well after a run, various alignments must be correct or the basic theory of the watt balance is violated. The square of the alignment angles enter as first order errors, so we need alignment accuracies within 0.5 mrad for a maximum of 0.25 × 10^−6^ error contribution. Thus a series of alignment tests is the first thing performed, and periodically thereafter, before recording valid results. The series of eight procedures starts with aligning the magnetic field to be perpendicular with gravity so the electromagnetic force on the induction coil aligns with the mechanical force. Bubble levels are used initially, but the lasers are necessary for some alignment checks, so they must also be aligned with gravity. Since the induction coil has some freedom of motion in several axes, its orientation to the field must be adjusted to reduce any coupling that would cause horizontal motions and rotations. This also includes making the center of mass, electrical center, and optical measurement center coincident. The final three checks align the mass pan, the rotation control electrodes, and the laser detectors to reduce unwanted external jolts and forces on the coil and optimize the signals.

Detailed modeling and calculations of the alignment sensitivities can be found in Refs. [10] and [11]. We make the following alignments in this order: 1) Align the axis of the superconducting solenoid to vertical. 2) Make the superconductor and moving coil coaxial. 3) Adjust all six of the laser beams to vertical. 4) Align moving coil axis to vertical. 5) Adjust the electrical axis of the moving coil to be coaxial with the center of mass of the moving coil, including its support structure. 6) Make the mean axis of the three interferometers and the center of mass of the moving coil coaxial. 7) Adjust the mass pan pivot to the center of mass of the moving coil. 8) Adjust the gold electrodes to vertical and the band position to minimum torque. 9) Maximize the signal from the displacement laser detectors and set the position and tilt detectors to zero.

#### 3.1.1 Magnetic Field—Gravity Alignment

The first two alignment procedures align the superconductor angle to gravity (discussed in this section) and the displacement between the superconductor axis and the moving coil axis (discussed in Sec. 3.1.2) (Fig. 4). A mutual inductance measurement technique is used in both alignment procedures. The same technique as used to measure a solenoid’s field in the proton gyromagnetic ratio experiment [13] is used to measure a mutual inductance at dc that is independent of frequency. The technique employs a special current waveform that changes smoothly between two constant current states. This current is injected into the mutual inductance primary. The integral of the voltage on the secondary (the total flux change) and the current change are used to compute the mutual inductance. Integrating the relationship *V* = *M* d*I*/d*t* obtains

$$M=\int Vdt/(\int Idt)=V_{\text{avg}}/(I_{1}-I_{2}).$$ (12)

**Fig. 5:**
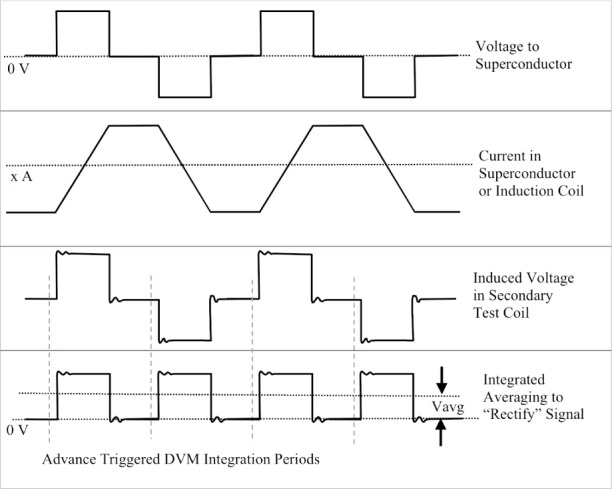
Waveform and triggering scheme used in making the mutual inductance measurement for magnetic field alignment. The resulting signal is insensitive to current levels and transition ripple.

Figure 5 shows the applied and resulting waveforms. Because the superconductor has a very large inductance, the upper voltage function in the diagram is used to generate a current in the solenoid following the second curve. When the moving coil is the primary, its inductance is much lower so we directly use the second waveform to generate the desired current. The output of the secondary is the derivative of this primary current and is shown in the third wave form. This output voltage is then synchronously rectified. In the earlier proton gyromagnetic ratio experiment, a lock-in amplifier rectified the signal. In this test, the pickup voltage signal is recorded by a digital voltmeter integrating at half the period of the waveform. Multiplying successive DVM integrals by +1 then −1 synchronously rectifies the signal, and averaging then gives the net flux in the coil as *V*_avg_, while eliminating any constant offsets. For a test frequency of 1 Hz the DVM has 30 power line cycle integration time, heavily rejecting the power line noise. The test frequency is required to be well below the superconductor’s low *Q* resonance frequency of about 30 Hz. Transients induced by sources such as ringing in the superconductor also average out by this method, as long as all transients decay to zero by the time the integration phase is complete and the integrating voltmeter is linear in its response to these transients. This is the power of this mutual inductance technique; it truly measures the dc mutual inductance. Checking that the output does not change with a phase change tests the measurement and analysis technique.

This first procedure also uses custom built equipment. A small probe coil, built with bubble levels attached, is placed in the superconductor’s magnetic field at approximately the same radius and height as the moving coil. As just described, the mutual inductance of the superconductor and the probe coil are measured and the probe angle adjusted to make the mutual inductance near zero. Then the bubble levels are set to zero as a reproducible mark. In an iterative, self leveling process, the probe is then moved to the opposite side around the superconductor. By keeping the levels at zero and the height the same, the angle of the superconductor is adjusted so that the same mutual inductance is achieved on both sides. Adjustment to the magnetic field is achieved by physically tipping the Dewar. By successively adjusting the superconductor, probe coil, and the bubble levels with respect to zero signal and probing several locations, the field is aligned as perpendicular to gravity. As a test, this probe was placed inside a level, precision solenoid (≅ 2.5 m long, 0.6 m diameter), to check the mutual inductance between the probe and solenoid. The probe signal was zero, verifying the method. The probe has a resolution of 0.2 mrad in sensing the field’s tilt angle, but an azimuthal asymmetry in the superconducting field limits the average alignment accuracy to about a 0.5 mrad. Fortunately, the induction coil angle alignment procedure (step 4) cancels any remaining angle misalignment.

#### 3.1.2 Induction Coil Alignment

The next procedure (step 2) positions the electromagnetic center of the moving coil relative to the solenoid using two mutual inductance measurements. The first mutual induction test is between the induction (primary) coil and a differential coil on a second probe station (shown on the left in Fig. 4), which gives a null reading when the probe is centered below the moving coil. The second test is between the superconductor (primary) and a secondary coil attached to the second probe station, but this probe coil is oriented at a right angle to measure the magnitude of the field. Because the superconducting field is radial, it falls off inversely as the distance from the solenoid axis. Hanging from the balance wheel, the induction coil’s position is changed by moving the balance translation table until the position of the moving coil results in the same reading from the field-sensing coil for all angular positions. This measurement was limited to 0.1 mm resolution.

Further coil alignments are performed with measurements from the laser position sensors. To adjust the lasers to vertical (Step 3), a plate mirror is leveled using a precision spirit level. An autocollimator is used to transfer the level to a small, flat mirror probe on a leveling assembly. Two spirit levels on the leveling assemble are adjusted to read zero when its mirror is level. This level mirror probe can then be used to make all the lasers vertical by autocollimation of the reflected light.

For the next series of alignments (steps 4 and 5), the balance is arrested to restrain any *Z*-axis movement. The *X*–*Y* position and tilt of the induction coil are noted with a 20 mA current applied. When the current is reversed, the coil’s horizontal displacement and angle changes are recorded. An unleveled coil causes an *X*–*Y* displacement. To adjust the moving coil angle we had to loosen, move, then retighten U-bolts that comprised the constraints between the spider and the three rods holding the moving coil. Once this alignment was close, further small adjustments were difficult to make, and accidental bumping of the coil during normal operation could change this alignment and was one limitation to the long term stability. Coil tilts from current reversals are the result of a center of mass (CM) (See Fig. 2) offset from the electromagnetic center. Angle changes were minimized by redistributing the CM of the coil via placement of brass slugs. After each change in the mass distribution, the moving coil must be moved to re-center to the superconductor, as recorded by horizontal displacement readings of the *X*–*Y* laser system.

For alignment step 6, the radius arms of the three corner cubes must be mathematically combined to equate the optical center (OC) with the CM, thus eliminating an Abbé offset error. A test procedure and program was written to find fit parameters for the OC calculation. This step was done on a regular basis, and always after a laser alignment or change in the mass distribution on the induction coil. In the procedure, the coil is purposely set to swing along the N/S or, in turn, the E/W axis. The interferometers are immune to side-way motion, but reveal the resulting tilt in the coil when combined appropriately. The three interferometer signals and knowledge of the dimensions of the coil permit calculation, with two fit parameters, of orthogonal motion for the *z*-axis CM and the two tilt directions. A series of interferometer signals is measured for swings as large as the beam detectors allow, then mathematically truncated to an exact number of periods. The fit parameters are mathematically varied and chosen so as to eliminate any common, in-phase motion between the tilt angle rotation and *z*-axis displacement, completing the orthogonalization. These two parameters are automatically incorporated into all subsequent position *z* calculations.

#### 3.1.3 Centering Internal Force

Step 7 is the alignment of the mass pan support relative to a vertical line through the CM of the moving coil. This eliminates any steady-state torque on the coil suspension when the mass is on the pan. We adjust this while the balance is arrested, by minimizing any horizontal displacements when the mass is placed on the mass pan. Since the pan is hanging on a conical sleeve around a ball at the end of a rod screwed directly into the main spider, adjustment is accomplished by changing the length of the three coil support rods. This primarily rotates the spider, thus displacing the mass support. Because of the need to iterate back through the alignments 4 and 5 after changing the spider, this alignment step was not performed frequently, and was very limited in effectiveness.

The adjustment of the gold plates that are part of the azimuthal rotation control system is necessary because this control is active during all measurements. When vertical, the three plates provide only tangential forces to the suspension and coil. The plate verticality is tested in a manner similar to the laser alignments, since the gold plates are highly reflective. A temporary alignment mirror is set into a vertical plane using two mirrors set at 90°, then a beam is autocollimated between that mirror and the gold plates.

### 3.2 Direct Reference Standards

For good reason, many fundamental constant measurements are performed in national standards laboratories. Having direct access in establishing traceability to many other NIST divisions is vital, as “standard references” need to be monitored closely. This section describes the actual reference and instrument calibration techniques, and the reasons behind the Type B systematic error estimates, as well as some history about why they were done in the manner documented [14].

#### 3.2.1 Frequency

Of all references used, the easiest to access and most precise should be a frequency standard for the time interval analyzers and Josephson voltage standard frequency counter. However, this was a little more complicated than expected in the days prior to inexpensive GPS receivers. Initially, a cable carried a 10 MHz reference signal from a LORAN C locked crystal standard in the Volt facility a kilometer away. This frequency was used to tune an old, 2 MHz crystal that had a fairly large drift on the order of 10^−8^ per month. But when the cable failed, we found a custom-built 5 MHz, ultrastable crystal to supply a frequency distribution box. This crystal was periodically checked against a 10 MHz rubidium standard, available as a part of a gravimeter system and tuned to within 10^−9^. After some time, the rubidium standard was subsequently recalibrated, and a 2.3 × 10^−9^ offset was “rediscovered” in August 1997. The offset had been determined by the manufacturer earlier, and accounted for in the reference database of the gravimeter analysis routine, but it was not compensated in the crystal frequency reference. Also, though the short and long term drift rates of the both the crystal and the rubidium standards are very small, they were not regularly monitored, so any offset correction could not be easily justified in the watt data. Choosing 5 × 10^−9^ as the Type B frequency uncertainty covers these errors yet has minimal contribution to the error budget.

#### 3.2.2 Length

**Fig. 6:**
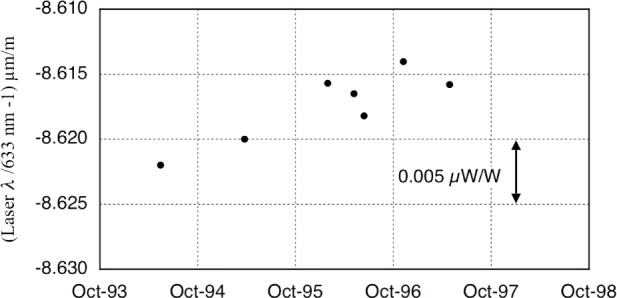
Calibration history of the wavelength of the He-Ne laser used for interferometry. An indicator bar relates the units of each figure to the equivalent fractional watt value.

Another reasonably simple calibration concerned the He-Ne laser frequency for use as the interferometry length standard. Figure 6 shows the record of wavelength checks of our laser against an infrequently borrowed, iodine-stabilized laser. Our He-Ne laser drifts about 2 nm/m/yr with about the same level of noise. Only the most recent calibration number was used in calculations. A Type B uncertainty component of 0.005 µm/m for this reference is the combination of the scatter along the linear fit to the drift plus the uncertainty calibration of a reference iodine laser.

#### 3.2.3 Resistance

**Fig. 7:**
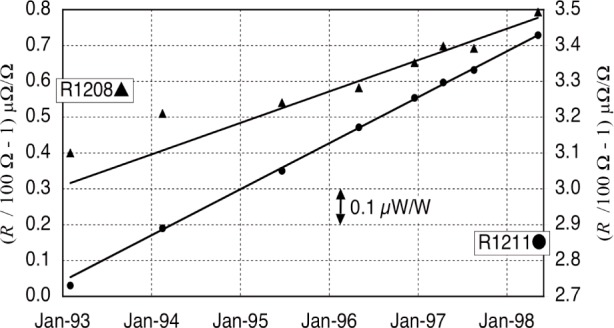
Calibration history of two resistors used in the experiment. R1211 was used as the main reference.

The reference calibrations now get progressively more complicated. Current, *I*, is measured as the voltage drop across a 100 Ω resistance standard. Figure 7 shows the history of the two resistors in the experiment, as they were periodically transferred to the NIST Resistance Group for calibration against a 100 Ω bank, traceable to a quantum Hall effect (QHE) system. Resistor R1211 was chosen for its low-noise characteristics as the main reference, even though R1208 had less drift and smaller temperature coefficients. R1208 was used as part of the constant current source, described below in the volt reference paragraph. Its value dropped out of the final calculations as long as it was stable over the hour between intermediate calibrations. Both are kept in a (25 ± 0.001) ºC oil bath. The last couple of transfer calibrations of R1211 were conducted directly against the QHE system. The Type B uncertainty components for this is 0.008 µΩ/Ω, and as with length, it is from the scatter along a linear fit to the drift plus the NIST calibration uncertainty.

#### 3.2.4 Mass

**Fig. 8:**
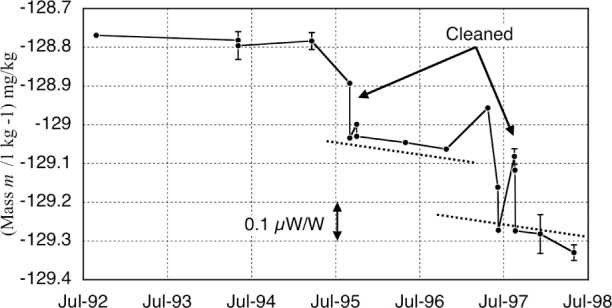
Calibration history of the 1 kg test mass. A few error bars (*k*=2) are shown for comparing the drift with the calibrations. Dotted lines have a constant slope to compare drift rates during times of heavy use. Sudden jumps are the result of cleaning the mass during calibration. Only the last segment was used in the value determination.

The test mass reference is two cylinders of gold totaling 1 kg. Figure 8 shows its history of calibrations performed by the NIST Mass Group. There are four reasons for its somewhat erratic behavior. (1) The Mass laboratory had been moved several times in the years leading up to 1998 and environmental conditions sometimes restricted calibrations to using steel masses, so the buoyancy mismatch added some uncertainty. (2) Gold is a soft metal, and with our experiment lifting the mass about 75 times a day, the cylinders showed signs of wear. The dotted lines on the graph have identical slopes and provide a gauge to suggest that the wear rates were similar during periods of continuous runs. (3) The mass resided during long time periods in the balance environment of unfiltered room air. Recalibrations done before and after cleaning the mass proved that the mass was accumulating dirt, so for better predictability, it was not cleaned during run periods. The combined drift from wear and surface contamination was assumed to be linear. (4) Several times the mass was dropped, usually from catastrophic loss of control in the balance. Some drops invariably led to extra mass loss and/or more dirt. All these added dings, scratches, and contact with dirty surfaces account for the larger shifts between some data groups. Even with a better calibration against the main NIST PtIr working standard mass and more careful handling, the intensive use of the gold mass in the final months (the last two points) increased its wear vs dirt accumulation, leading to increased uncertainty. A 0.02 mg/kg uncertainty contribution for the mass reference accounts for both the NIST calibration uncertainty and the possibility that the actual drift was not continuously linear, but could have changed rapidly after being transferred from the cleaner mass lab into our dirtier experiment environment, then drifted more slowly.

#### 3.2.5 Voltage

**Fig. 9:**
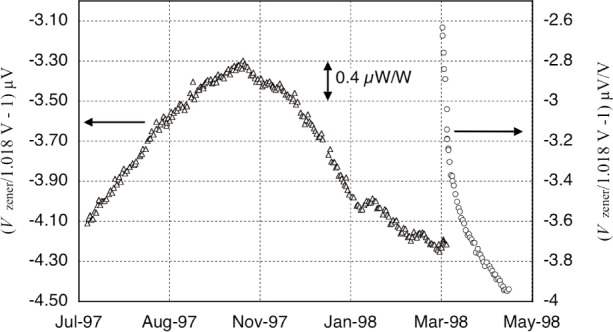
Calibration history of two Zener references at 1.018 V against a Josephson array volt standard. Although having a steeper slope, the Zener with the open circles is more accurate for day-to-day interpolation.

Finally we address the voltage uncertainty. Even though it is the third largest contribution in Table 1, it has an uncertainty that is excellent, considering it encompasses a three-step transfer at 1.018 V to establish traceability to a Josephson array voltage standard system, with an uncertainty of about 0.0025 µV/V of its own. The value 0.03 µW/W is the voltage contribution *to the watt*, which enters as *U*^2^. Voltage is referenced against both the coil’s generated voltage and the force-current across the resistor. The tertiary reference is a low-noise voltage source consisting of a Hg battery-stabilized current source and a 100 Ω resistor (R1208 mentioned previously). This source will be referred to as the Hg-reference. Its peak-to-peak noise over minutes was less than 10 nV, much less than a Zener reference, but the drift was typically 500 nV/h. This current/voltage source is calibrated hourly against a Zener reference during a watt run. Both of these sources were kept in a corner of the balance room where temperature was generally controlled to (22 ± 0.05) °C. A Josephson array voltage standard system was set up in the same building to calibrate the Zener reference daily. Figure 9 compares the history of two Zener references used in the later months of the experiment. The data includes corrections for thermal emfs (typically about 100 nV) in the 10 m leads extending to the Zener. Although the second Zener was suffering an extended nonlinear drift, a “burn-in” from a recent repair, it had less noise and better linearly-modeled drift over the short term of several days. The voltage uncertainty assignment is a combination of the residual errors from the short term linear fits, the scatter in the thermal emf of the Josephson system to Zener connecting wires, and a small fraction more for the Josephson system itself.

### 3.3 Correction Factors

Quite different from the direct reference standards in the main balance system, there are three independent measurement systems that determine essential variables used as correction factors to the main measurements of force (acceleration of gravity and air buoyancy) and velocity (air refractive index).

#### 3.3.1 Acceleration of Gravity

**Fig. 10:**
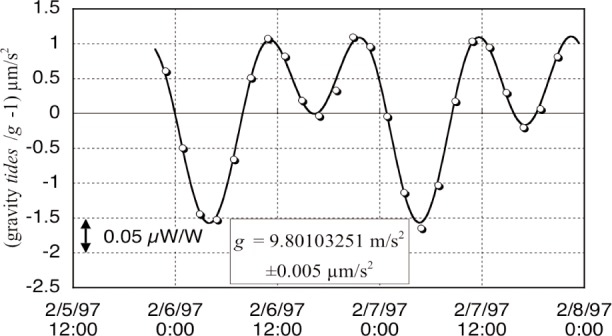
Gravimeter data. Open points are averages of 100 drops in 17 min, offset from an averaged value for *g*. The solid curve is the calculated tidal variation. The uncertainty of 0.005 µ/W represents the residuals for this fitted tides calculation.

We examine the gravity determination first. Gravity acceleration, *g*, is determined by a commercial gravimeter [15], which records the trajectory of a corner cube dropped in a vacuum. Figure 10 shows a graph for points from a typical measurement run. The continuous curve is from the tidal effects calculated from U.S. Geological Survey (USGS) software [16] for our location, and must be subtracted from the data to obtain the local average, 9.80101933 m/s^2^. (These same tides are calculated as corrections to the hourly forces recorded during watt runs.) The scatter in the open circle points may be from other diurnal effects related to tides that cannot be computed, but are small enough for us to ignore. The (tide corrected) *g* value for our locality is assumed constant, since lower resolution checks in previous decades gave about the same result. To establish traceability, in the summer of 1997 the gravimeter’s manufacturer recalibrated the iodine-stabilized laser length standard and rubidium frequency standard and the atmospheric pressure meter, needed to estimate the air mass overhead. This check verified the system’s Type B relative uncertainty specification of 2 × 10^−9^, and obtained random uncertainties of the same magnitude. An additional gravity correction is necessary for the actual mass position at 4 m higher and about 5 m away from the location of the gravimeter. A USGS team determined this with a gravity transfer meter in Sept. 1997. Set up near the upper cabinet and as close to the mass as possible, they obtained a fractional correction of (−1.363 ± 0.005) × 10^−6^. This is reasonably consistent with a gradient calculated from the gravimeter. The combination of these system errors is a Type B contribution to watt error budget of about 7 nW/W.

#### 3.3.2 Air Effects: Refractive Index and Buoyancy

**Fig. 11:**
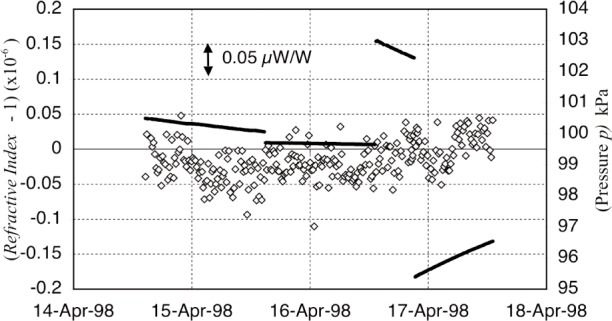
Refractometer data. Open points are the difference between the measured and calculated refractive index of air. Dark lines are the pressure, proportional to the index = 263 × 10^−6^ at 100 kPa (25 °C, 40 % RH).

In contrast, the refractive index and the mass buoyancy in air are continually calculated from measurements of temperature, pressure, relative humidity, CO_2_ concentration, and other air constituents. The calculated corrections for the refractive index and the buoyancy effect on gold are about 250 µW/W and 60 µW/W respectively. Monitoring the environment parameters for these calculations [17] required a large number of sensors. The dominant parameter in both is pressure. A single sensor at the level of the mass recorded pressure for the buoyancy correction, but with the induction coil 2.6 m lower, 0.2 Pa was added for the refractive index calculation (about 0.5 µW/W). This offset was both calculated and checked with a second pressure meter, which also acted as our transfer standard to the NIST Pressure Group. Four thermistors were used to measure temperatures at locations near each of the interferometer paths and the mass. These thermistors were calibrated with an ac resistance bridge thermometer, which in turn was calibrated by the NIST Thermometry Group. We calibrated a relative humidity sensor, which could be easily read by computer, against an absolute dew-point hygrometer. A CO_2_ meter was used on occasion, registering an average level fraction of 400 × 10^−6^ in the balance room (and that it rose significantly when people were working inside). A difference of 100 × 10^−6^ meant a change of only 0.0025 × 10^−6^ in buoyancy so we relied on its manufacturer’s spec. Several tests with a residual gas analyzer showed no large concentrations of non-standard gasses, especially helium, at least for the final data group. There were occasional helium leaks that did occur and gave measurable offsets in several sets of data. All these measurements and their application into the calculations for the refractive index of air were checked with a dual path interferometer, built by Fujii [18] and improved by Newell. Figure 11 shows a set of measurements agreeing with calculations to within 0.05 µW/W, which was consistent with the combined uncertainty of all the sensor calibration uncertainties.

A significant effort went into controlling the temperature of the balance room and reducing gradients, especially the large ones of several degrees due to proximity of the liquid helium Dewar. One complete control system regulated the two-story room, monitoring the temperatures at eight different locations over five elevations and inside the lower box surrounding the induction coils. Non-inductively wound ribbon cable was used as heater tape, and powered by 110 V ac. The ribbons ran in parallel strips at five levels from floor to ceiling, with the thermistors located just below the level of the ribbon. SCR devices switched current into the tapes in a time-division pulse train, synchronized with zero crossings of the line voltage. The control point was about 22 °C with a positive gradient of 1 °C from floor to ceiling to minimize air convection. Normal variations are about 20 mK/h, with a several degree excursion for several hours after a liquid helium transfer. A plastic sheet impregnated with wires was wrapped around the Dewar at the level of the lower box. These wires were connected non-inductively in a vertical alignment to act as a heater strip. A ninth thermistor was placed between the box and the heater strip to set the temperature near that of the room temperature, thus lowering the gradient across the induction coil and the laser interferometer paths.

A study into the temperature gradients near the mass found that the base of the upper box was several kelvins colder than the top of the box. To remedy this, we adjusted the temperature of a flat heater cord wound around the helium gas exhaust line, just beneath the box. This raised the box floor to about room temperature. Additionally, the mass mover arm was thermally isolated from the mass, since it’s lower cantilever arm was close to the cold box floor and was conducting heat away from the mass far faster than when the mass was on the pan. After these changes, the thermal gradient for about 5 cm around the mass was reduced to less than 5 mK.

### 3.4 Induced Noise Sources

#### 3.4.1 Vibrations

**Fig. 12:**
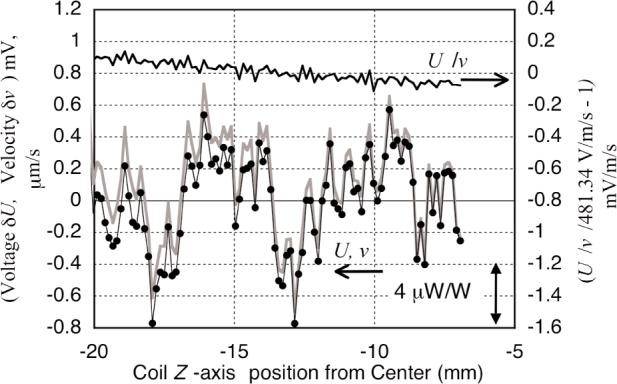
Velocity and voltage mode data. The gray line connects points plotted as the deviation, δ*U*, from an average 1.018 V. The closed points are similarly, δ*v*, velocity deviation from average of 2.11 mm/s. The jagged line at top shows the noise reduction in the δ*U*/*v* ratio, where both *Y*-axis scales are equal. The δ*U*/*v* slope arises from the *Z*-dependence in the magnetic flux density.

Measurement noise, as opposed to transfer uncertainties, limits the precision that can be obtained in a single run for both modes of the balance. This is a brief description of the effects of three of the known noise sources. The largest noise source, but not a decidedly systematic contribution, is vibration. The largest source of vibrations is the building ventilation fans. Even though located some 80 m away in a separate building, the vibrations apparently carry quite well through the ground surface. Located at about 150 m is the cooling tower for the NIST Cold Neutron reactor. Vibrations enter the coil support assembly via a structural vibration mode resonance of 28 Hz. Vibrations in themselves should not be a problem, since motions relative to the fixed inductive coil with velocity *v* correlate with voltage *U*. The ratio of these variables is a function only of the magnetic flux, but the ratio does have a large remnant of 28 Hz noise. The individual *U* and *v* noise and *U*/*v* ratio signal can be seen in Fig. 12. Though not as good as desired or expected, note that most of the large fluctuations track equally, so that the noise is reduced by about a factor of 70, with about 20 µW/W peak-peak noise remaining. The small slope of the ratio is due to the varying magnetic flux as the coil moves along the *z*-axis. An initial suspect for the residual noise in the ratio was the low tank circuit (R-L-C) resonance for the coils, which did cause some phase delay of 28 Hz voltage. A small effect was also assumed to come from acoustic waves generated from the floor, walls, or the coil itself. These would register across the interferometer beams as small velocity fluctuations with no corresponding voltage. (Only recent studies of the coil’s mechanical characteristics revealed that the 28 Hz resonance was a flexing mode of the coil, which caused the interferometers to move out of phase with respect to the average motion of the coil.) As far as we determined, this type of noise contributed a random error. Although we made most runs at night in the winter with the fans turned off, there was no change in the watt results when the fans were on and the velocity data was noisier.

#### 3.4.2 Electromagnetic Interference (EMI)

Another noise source is EMI, mainly at multiples of power line frequencies. The series-opposition wiring of the fixed coil effectively cancels 60 Hz, but higher harmonics of 360 Hz and 480 Hz arise from slight variations in the R-L-C resonance of each coil. These are filtered with the signal integrated at power line frequencies. A chopper-stabilized pre-amplifier was sometimes used for zero-field testing, but because these noise frequencies were close to the sampling frequency of the amplifier, large amplitude signals arose at low beat frequencies. We also found that some high frequency (MHz) EMI interfered with the signal output of the interferometer or *x*-*y* position laser detectors. The temperature sensor circuits were also very sensitive, and could read temperatures incorrectly. Possible sources of higher frequency EMI noise, (computers, CRTs, etc.) were kept as far away from the detectors and coil as possible. An error estimate of the possible EMI effects is about 0.01 µW/W.

Different circuit connection schemes were tested to find the best noise rejection. The signal lines were routed through twisted-pair, low thermal cable consisting of silver-plated copper wire, with Teflon insulation and a braided shield. Interconnections were thick copper lugs, soldered with either low thermal Cd-based solder, or more recently, eutectic Pb-Sn solder. Crimp connects were also used. The copper lugs were cleaned every few months. Leakage resistance to ground was checked. Several bad multi-wire connectors leading to the induction coil were found and isolated from ground using Teflon tape. Leakage resistance was measured as at least 10 GΩ between several locations and ground. Leakage between wires in the bad connectors did put much lower resistance in parallel with the coil, which theoretically should not matter, but this was tested. Comparing normal data with a set taken with a 10 MΩ resistor in parallel with the induction coil showed that the voltage error contributes inversely in velocity and force mode, so that the watt value remained the same. It is difficult to calculate the effects of all the possible leakage paths so we modeled the leakage resistance to ground as a simple parallel current path to the coil, taking the worse case leakage measurement. This gives a maximum leakage resistance error estimate of about 0.02 µW/W.

#### 3.4.3 Knife-Edge Hysteresis

**Fig. 13:**
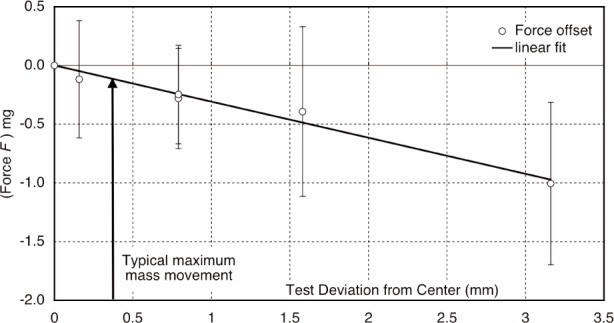
Knife edge hysteresis test. After rotating the 30 cm radius wheel (linearly moving the mass pan) for 1 minute, the change in the balancing force upon return to the previous postion was recorded. The arrow indicates typical, absolute value of maximum offset during mass placement. Because of the varying sign of position movements, hysteresis effects contributed mostly random force offsets. The error bars are the standard deviation quickly repeated trials, and their size suggests that this is the dominant noise source in force measurements.

Excess balance motion results in noise or systematic errors in the force mode, because they induce knife-edge hysteresis. A time dependent restoring force was first noticed in special force profile measurements (previously used to determine the field profile, but only used as a check by the end), where force balance measurements where made at different locations away from the usual center. This hysteretic force is assumed to result from induced distortion in the edge geometry after rotation. To quantify the knife-edge hysteretic effect, we recorded the balance current at the normal position then made small rotations, varied with time and amplitude. When returned to the previous balance position, this rotation clearly produced time-dependent restoring forces roughly proportional to the rotation amplitude, as seen in Fig. 13. Other tests indicated a similar effect for time duration off center.

In a test to try erasing the knife-edge hysteretic effects, the balance was intentionally rotated up and down during a run by about 800 µm after the mass was positioned. The result was a surprising failure; the force current noise increased tremendously. So to study the actual behavior of the balance, the motion of the wheel was recorded during the mass placement for the final weeks of the experiment. Several typical behaviors were observed. As mentioned above, there is usually a brief time while the balance maintains some amount of offset from zero position, but with the servo-control underdamped, it recovers its position in a decaying oscillation. The maximum offset is reliably reproduced over a long progression of placements, but the direction varies between runs that are days apart. To estimate the effect of this balance motion, the amplitude curve of each trace was numerically integrated relative to the excursion time. Each trace usually had a net integral offset value, however, and most significantly, the integrals averaged for two weeks worth of well-controlled runs, counting about 1400 force measurements, was nearly zero. As a result, the Type B uncertainty for the knife edge hysteresis was estimated at 0.02 µW/W, corresponding to the force offset for a typical maximum balance excursion of 200 µm before a single weighing. With typically small deviations over the long term and the conscious elimination of larger excursions flagged by extreme noise or clearly out-of-control runs, we assume most of the hysteresis effects of typical short term rotations randomly average out.

## 4. Watt Data Acquisition

There are three distinct measurement modes in the operation of the watt balance experiment: 1) environment calibration, 2) volt/velocity ratio, and 3) force/current ratio. A single point consists of the progression 1-2-1-3-1-2-1. For multiple point runs, the pattern is repeated from 3 on. Although the programming details were modified and refined over the years, this measurement strategy was maintained. The programming of this was originally done in BASIC in the late 80s, reviewed and restructured in 1993–4, and totally rewritten in an icon-based, graphical user interface, programming language in 1996 for a newer generation of computers. These recent programs were written to be largely reusable, both as routines available for quickly designing special tests and for the future versions of the experiment. It is beyond the scope of this paper to describe all the programming details and tricks, but part of the improvement to this version of the experiment must be credited to the modernization of the computers, signal acquisition and analysis programming, and data analysis software. Plus the speed and flexibility allowed us to modify the procedures, reanalyze and view data, or design special tests more quickly.

### 4.1 Calibration Mode

Between each ratio mode, all the variables are measured for applying the voltage and environmental corrections. To establish the short term voltage drift of the Hg-ref, the voltage difference between it and the Zener is recorded in four pairs, measuring both polarities of the current reversing Hg-reference against the switch = reversed Zener. The standard uncertainty of this measurement was about 0.02 µV. The pressure, relative humidity, and temperatures were measured near the regions of: the laser paths at the induction coil, the mass, and the resistor in oil bath. Other temperatures around the experiment were also monitored. Recording all the sensor readings takes about 5 min, with most of the time spent on the Hg-reference voltage check. In post-experiment analysis, the individual variables were extracted in pairs of two for linear interpolation, satisfying most of the slow temperature drift. Since the Hg-reference voltage tended to change nonlinearly, more points were extracted for fitting to a 2nd order polynomial. We found air pressures could change quickly and irregularly, resulting in large effects on the refractive index and buoyancy corrections, so we began measuring pressures immediately before and after each velocity and force trace. A linear drift rate between these two readings was then applied to the proper calculation.

### 4.2 Volt/Velocity Ratio Mode

#### 4.2.1 Velocity Control

In the *volt/velocity* mode, the magnetic flux must be determined at the precise coil position where the test mass is balanced in the *force/current* mode. In this velocity mode, there are two data acquisitions to describe. One is for measuring the voltage signal and the other to measure coil position and time signal. All signal voltages are measured as a difference against the Hg-reference voltage source, using three DVMs with inputs connected in parallel to the series opposition circuit of the induction coil and reference. A fourth DVM with separate timing is used to servo-control the coil velocity to the constant voltage of the same Hg-reference. A simple lead-lag feedback routine digitally controls a feedback current to the countermass electromagnet. This control was complicated because the feedback signal reflected the large, vibration-induced voltage noise at 15 Hz to 30 Hz, near the desired feedback bandwidth. This limited the feedback frequency. Also, the gain of the feedback has to be low to insure a total gain less than unity at the mechanical resonance. A high level of control is achieved by using a “learning” technique. The computer records on each velocity pass the direct feedback digital/analog converter output relative to coil position, obtained via the upper laser. This coarse feedback trace for each direction of travel is digitally filtered and integrated with previous passes, then applied as an offset to the direct low gain feedback signal on the following passes. In this way, both short term balance wheel diameter irregularities and long term changes from a drifting voltage reference are compensated. This voltage control routine maintains an average voltage difference of less than ±1 mV. For us, controlling to constant voltage proved better than controlling to constant velocity. Integration capabilities of the DVM automatically filtered some of the large vibration noise (tens of mV (µm) at 28 Hz) that the low resolution of 0.025 *λ* and slow readout of the upper laser interferometer could not handle. This method also minimizes voltage gain errors in the DVMs. To minimize coil *X*–*Y* motion buildup and accompanying Abbé offset interferometer errors, the swinging of the coils is actively damped between each pass.

#### 4.2.2 Velocity and Voltage Data Acquisition

Measurements of the changing position of the induction coil are made with a heterodyne interferometer. The dual polarization beam is from a thermally stabilized HeNe laser (633 nm). With the fixed coil as reference plane, uniform vertical motion of the system as a whole does not register, since it is common motion to both coils. Two different fringe detectors were tested. A commercial detector was quieter but had a lens causing possible shear errors between the two polarized beams (a problem when the coils swings). A custom-built, lens free, detector was eventually adopted, opting for less possible shear error but slightly more electronic noise. Three interferometers (see Fig. 2) call for three, dual channel, time interval analyzers (TIA) to record the phase relation against the 1.6 MHz reference signal and time readings, for up to 4096 position-time readings. The TIA resolution is about 1/250 fringe, although the detector noise floor is more like 1/25 fringe. A velocity at about 2 mm/s is achieved for 7 cm total travel, recording data for about 40 s. At this velocity, the *λ*/2 optical fringe-crossing frequency is about 6 kHz. To minimize the possibility of nonlinearity errors due to polarization mixing, each TIA records multiple readings per trigger, timed to equal one fringing period (see Fig. 13). These group readings are programmed for equal timing over the approximate interval that one fringe passes (≈158 µs). This minimizes polarization nonlinearities, which average out over each fringe cycle [19]. As mentioned above, since the magnetic flux profile is not constant, controlling velocity to a constant voltage does imply a slightly varying velocity, so there is a very slightly higher potential for polarization mixing errors. The calculated average of the group gives a position and time reading. As mentioned earlier, the three interferometer signals are mathematically combined with Abbé corrections to locate the coil center, compensating for any angular tilt of the coil. The average velocity is calculated from the difference between successive position and time determinations.

**Fig. 14:**
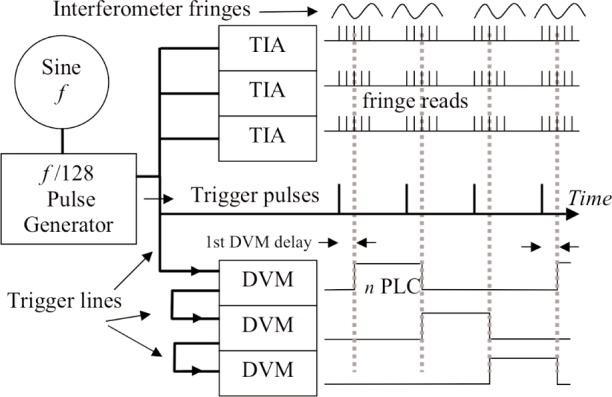
Wiring and timing scheme for applying pulse triggers to the instruments. The dashed lines indicate the recorded timing coincidence between the DVM integrations and the averaged TIA measurements. Note that the TIA measurements cover very nearly one interferometer fringeing period to compensate for polarization mixing effects.

To facilitate timing accuracy, the voltage signal is recorded with high speed, precision DVMs. The recorded voltage is the difference between any induced voltage of the moving coil and the fixed coil, minus the voltage reference. In this way, 60 Hz V ac noise and vibrations of the magnet solenoid are heavily cancelled. A DVM auto-zero function for each individual measurement is incorporated to reduce internal DVM noise. However, the additional auto-zero gate time plus DVM processor time is just a bit more than twice the data integration interval, so continuous voltage recording requires three DVMs. Modeling showed that minimizing the dead time greatly enhanced high order curve fitting. So to record with very little dead time and synchronize with the TIAs, we developed a triggering scheme (Fig. 14) to time each of the three interleaved DVMs with all of the three TIAs.

#### 4.2.3 Instrument Triggering Scheme

The basic scheme is as follows. First, sharp trigger pulses are generated at integer periods with respect to the nominal 60 Hz power line cycle (1/60 s = 1 PLC) with timing that is variably set with a programmable frequency synthesizer. The phase jitter of synthesized square wave edges is reduced by running the synthesizer at higher frequencies and generating the actual trigger pulses with a 1/128 dividing circuit. The synthesizer frequency is set at the beginning of each velocity pass. Since the divider cannot be reset, the initial trigger cannot be synchronized to a specific position. When pulses do start, the first pulse triggers the TIAs and DVM 1 integration begins. For the multiple TIA group recordings, the internal delay on DVM 1 is set for a time at the middle of the TIA group reading. DVM 2 is programmed and wired to trigger upon the end of the gate time of DVM 1, which corresponds to the next trigger pulse that has triggered the TIAs plus delay time in DVM 1. While DVM 2 integrates, DVM 1 now enters its auto-zero mode and does not register this trigger. Similarly, DVM 3 is triggered by the end gate time of DVM 2. DVM 1 is still processing data, so does not react to this third trigger pulse. At the fourth trigger pulse, DVM 1 is again ready to respond, and the cycle repeats.

Considerable time was spent studying different integration times in order to minimize both the noise sources of 60 Hz V ac and the background vibration noise at 28 Hz. Since the fixed and moving coils were not perfectly matched, the 60 Hz signal was about 30 mV peak. The DVM integration timing is normally set as an integer number of PLC. However, the DVMs used here do not maintain measurement aperture timing directly off the power line zero crossings, but by estimating a line frequency timed by intervals of its 50 MHz internal clock chip. The local power line frequency drifts on the order of milliHertz per minute. This drift means that there is almost always a phase error between the power line frequency and a “constant” DVM timing preset for exactly 60 Hz. Although the DMV can be set for triggering to a line frequency, zero crossing the TIAs also need to be triggered. Simulations showed that we could minimize this phase error to no more than that accumulated in 40 s and also synchronize the TIAs by using the DVMs to read the line frequency and presetting the DVM integration aperture the proper period immediately before a velocity pass. Then both the DVM gate aperture time and trigger pulse timing are set to match. Thus, each velocity run starts with nearly exact line frequency matching. The usual period was set for 4 PLCs, since was roughly twice the period of the 28 Hz vibrations, further reducing this source. There is a small timing discrepancy inherent in this scheme. Since the DVM relies on its internal computer clock cycle to respond to triggers, the assumed start and stop times of the DVM integration periods are only accurate within a 0 ns to 200 ns range.

The DVMs are calibrated often to minimize some possible voltage reference errors. The internal frequency reference is calibrated at 100 kHz so the 60 Hz line frequency is measured as precisely as possible. Self-calibrations were performed on the DVMs to minimize apparent noise in the signal from offset errors between the three DVMs. The three main DVMs were chosen out of those on hand for minimal offset drift. The gain error was checked and calibrated at 1 V against a Zener reference calibrated *in situ* by a Josephson voltage standard. The 1 V range was used for the velocity signal measurements to prevent DVM overloads from occasional voltage noise or spikes greater than 100 mV. The increased resolution of the 0.1 V range was available for other measurements: force and Hg-ref to Zener calibration.

At the end of each velocity pass, the raw data from all three TIAs are not stored. Due to lack of computer disk memory at the time, each TIA’s fringe group counts and times are averaged, and then the three interferometers are combined and stored as a single coil displacement point. Even with this compression, a typical nightly run contains about 1 MB of total data. The data from the three DVMs are interlaced into one continuous trace, coinciding with the fringe and time array elements from the TIAs.

### 4.3 Force/Current Ratio Mode

#### 4.3.1 Control and Current Data Acquisition

**Fig. 15:**
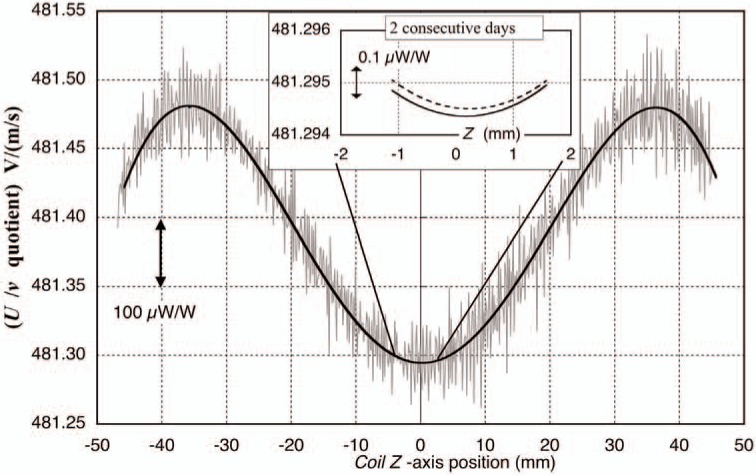
The response profile of the induction coil to the magnetic flux density, shown as the voltage/velocity quotient. The inset expands the center to show the slight daily variations of the relative position of the local minimum. Sampling time is 15 Hz.

The current measurements in this mode are relatively simple, although the details and subsequent noise and error sources are not. The balance weighing position is always the same, measured by and servo-controlled against the position signal from the upper, axially aligned interferometer attached to the main support spider but separate from the mass pan. The force of the mass is balanced within the support structure, so the tension on the bands is constant, and thus the balance position, i.e., the knife edge angle, is highly repeatable. The same lag-lead subroutine as used in velocity control but with modified control parameters, maintains the balance position within about 300 nm. The balance *z*-position is chosen to be at the zero slope “center” of the magnetic flux profile, derived from analysis of the velocity mode data (Fig. 15). The curvature in this vicinity is about 0.02 × 10^−6^ over ±100 µm from dead center. A 0.5 kg tare mass is placed onto the countermass side, and servo-balanced with −10 mA of current, which also passes through a 100 Ω reference resistor. The voltage drop across the resistor is recorded in the same 3 DVM scheme as in the velocity mode. A balance trace lasts about 2 minutes, recorded at 60 PLC integration time per reading. This first step represents a zero mass value, since in the next step the 1 kg reference mass is placed on the main mass side of the balance. This force is counteracted with +10 mA current. The symmetry of this procedure cancels any voltage offsets and maintains a constant coil temperature rise due to joule heating. The 1 kg mass is placed on and off for typically six pairs of readings. The coil tends to swing quite a bit with mass placement, so the *X*–*Y* swinging is actively damped until the measurements recording starts. A typical force mode lasts a half hour, roughly matching that of the velocity mode.

#### 4.3.2 Mass Placement

These readings (and those from the velocity mode) are susceptible to several noise-inducing effects that may also cause systematic errors. One is a disruption of the magnetic field current control. Occasional random current spikes occur, caused by RF noise or sudden pressure changes in the liquid helium system. As a smaller but potentially worse effect, any alignment mismatch or off center position between the induction coil and the magnet also results in a non-zero mutual inductance. In this case, the magnet current control reflects any sudden change in the pickup coil current and takes a few tens of seconds to recover. If the mismatch is large enough, as occurs for large off center excursions in a force measurement, the magnet current might not have recovered by the time the force readings are triggered. Program intercommunication was not developed enough to correlate both data sets in real time, but noise spikes were recorded, and these were checked to identify if they correlated with force current discrepancies. These could then be considered as non-statistical outliers and discarded. An effect that was easy to see, but impossible to control, affected only the first few force measurements. If the tare mass had not been properly pre-centered, it could hit its mass pan off center and cause the countermass to swing. Without swing damping on that side, it takes several minutes to damp out from the drag of two thin, silver chains connecting the countermass to a nearby post. The current servo’s struggle against this slowly oscillating force is revealed in a 10 s period waveform in the recordings.

Although the force mode is described as a static measurement, small wheel rotational motions occur during the complicated routine of shifting the mass onto or off of the pan, and these affect the later measurements. Normal, but imperfect, physical dynamics and a rather coarse mass placement scheme is the cause of this motion. The upper interferometer is read at about 20 ms intervals, but the mover arm’s position is recorded as a worm-gear screw rotates in tandem with a resistor potentiometer. The change of resistance was calibrated against the actual motion (via the interferometer) of the mover, but precise motion control was not very reproducible and the DVM used in reading the pot resistance was slow. Excess balance motion occurs because this timing interval for reading the positions of both the mass and the pan was only 70 ms, and with changes in the potentiometer *z*(*R*) calibration (from humidity, DVM offset, etc.) the position of the mass relative to the pan is not known to better than 100 µm. There are also only two speeds hard wired into the worm gear drive electronics, fast for initial approach and slow for final landing.

As the mass transfers from the cantilever holding arm onto the pan (noted as a change in the interferometer reading), the elastic response of the arm adds to the motion of the mass, pan, and wheel. To compensate and give the servo control time to adapt, the arm is moved and stopped several times after the mass hits the pan. The typical motion resulting from this routine is an initial pan displacement of about 200 µm (0.6 mrad rotation) lasting about 10 s. Although the balance servo initially moves in the direction of motion of the mass, the balance usually overcompensates a greater amount in the opposite direction because of differences in the levels of the arm’s three contact pads relative to those on the pan. Depending on the timing of the motion sensing routine, another displacement occurs, mostly less, sometimes greater, and in unpredictable directions for another 10 s. Finally, the arm is moved out of the way and the servo recovers with under damped oscillations over another 20 s. This motion was noted during programming and later recorded toward the end of the experiment. Very poor mass handling behavior could be noticed in the daily watt data. For a brief number of days during the months of data acquisition, the force current values were noticeably noisy and the watt values clearly different from the long term average of the data. The knife edge motion during these runs correlated to a peak balance motion of 1.2 mm (4 mrad) or more, and further investigation found a severe misalignment in the mass mover system, degrading the mass controller’s performance. As a result, mass mover alignments were done every week or two to minimize the excess balance motion in this mode.

## 5. Watt Data Analysis

A separate program was written to analyze a complete run of recorded data. It was continuously modified for mistakes as well as improvements, and many runs were processed several times, sometimes just to test the validity of the different analysis methods. This section describes these methods.

### 5.1 Volt/Velocity Ratio Analysis Programming

#### 5.1.1 Basic Analysis Description

Post-experiment analysis starts with retrieval of the runtime calibration data, the stored voltage/velocity data, the stored force/current data, and a file containing the basic reference data: laser wavelength, mass, gravity acceleration, Zener voltage and drift slope, and resistor value with slope and temperature coefficients. For the velocity data, the fringe counts are converted into meter units by applying the laser reference length, corrected for the refractive index based on the environmental readings. The velocity is calculated by taking the difference between consecutive positions and times. An average time and position is assigned to correspond with each average velocity and integrated voltage difference. The voltage differences are added appropriately to the volt reference. Then a voltage/velocity ratio is calculated for each point along the length of travel. The recorded, pre-analyzed, data can be graphically displayed during the analysis for visual inspection, which often helps in later evaluating the performance characteristics or identifying intermittent problems.

#### 5.1.2 Early Analysis Methods

Although the only volt/velocity ratio needed is at the center force balance position, using only one point would be far too imprecise, given limited spatial and timing resolution. Gaining greater precision by averaging over more points away from the center is complicated, since the magnetic flux profile is a curve without a simple equation representation, varying by about 350 × 10^−6^ (Fig. 15). Simple averaging or linear fitting of the trace data to evaluate the center point is obviously insufficient. Historically, a profile model was constructed from force measurements at various positions up and down the *z*-range, because the force measurements were much quieter than the velocity measurements. Off center force measurements were bracketed by, and normalized to, measurements at the center in an effort to compensate for the unknown changes in the magnetic field and other corrections. The resulting 6th order simple polynomial fit was then applied as a correction curve to the velocities for months at a time. Sufficient for a previous watt determination [8], we realized that this profile was too variable. Although the curvature of the profile was fairly constant, its absolute relation to the *z*-axis could change slightly over days (Fig. 15 inset), and a single curve model became less representative as the skew current source drifted to cause asymmetry. So we came up with a scheme to obtain a better approximation of the curve from the noisy velocity data that would be up-to-date for each day’s run.

This new velocity data analysis has two goals, 1) to increase the signal-to-noise ratio by averaging multiple traces, and 2) to do a curve fit that uses as much *z*-axis data as possible to establish the ratio of voltage/velocity value at just one position of the force balance. It normally helps to average over as many points as possible to decrease the effect of noise. The 1.018 V signal, at 2 mm/s velocity, could be maintained for about 7 cm of the available 10 cm clearance. By integrating the DVMs over 4 PLC, the 15 Hz sampling helps to reject the dominant 28 Hz noise. Each pass consists of about 600 points. Three variations of an analysis method were tried, all proving reasonably equivalent over the long term. In one method, the data for each pass is fitted to an 8th order Chebychev orthogonal polynomial using a Forsythe [20] iterative scheme. Sixth and eighth order fits proved adequate to model the gross curvature without producing short range oscillations in the data; 8th was generally used. The coefficients are then used to calculate the value at the center weighing position. This simple method works, but the interpolated values for the center position are most heavily influenced by the nearby positional data, while the extremes have less weight. Also, occasional noise spikes can influence the curve fit.

A more complicated, two step method was adopted to improve the signal processing by eliminating noise spikes yet compensate for slowly varying profile changes. The analysis of each daily run begins by fitting each data curve to an 8th order polynomial, usually 20 up and down passes per set with about 10 sets per nightly run. The iterative fitting routine is efficient in producing good fitting coefficients, but it is difficult to establish how reliable an average profile may be by simply averaging these coefficients. Instead, we averaged curves from each pass. In the first attempt at averaging multiple traces, all the raw velocity/voltage ratio curves are sorted by *z*-axis position into an array representing position bins, spaced at about 130 µm, as the DVM timing interval and coil speed dictated. The intent was to average out noise spikes within each position. The limitations to this method were that the start time and velocity variations smeared the data into the coarse bin sizes, and there were occasional extreme noise spikes or profile traces that clearly did not have the “standard” profile. These tended to overly influence the averaged curve, and from past experience, the “standard” profile would change over time. So, a more sophisticated scheme was designed.

#### 5.1.3 Improved Analysis Scheme

For the analysis routine that was ultimately adopted, we start with an temporary profile curve averaged from just the first velocity set. This is a gauge curve for a statistical evaluation of all the succeeding curves, which helps filter out bad traces. Data sets deviating from this gauge curve above an established minimum standard deviation (or from a historical curve, for the gauge curve itself) are eliminated from the overall averaging routine, usually from excessive noise bursts or other non-typical profile behavior. In place of the bin-averaging scheme, a reconstructed curve for each data trace is then segmented into intervals, and these averaged, fitted values are added into the array bins. This averaged curve, obtained from the total of all the velocity sets in the first analysis, is fitted to 8th order and normalized to equal 1 at the center force position. Then a second analysis pass is performed. This average profile value is treated as a proportional correction, arithmetically divided into each raw trace data. This linearizes each velocity data pass without adding an offset to the values, and preserves short term variations and long term drift. The result is 600 points looking like a flat, noisy line, normalized to the value of the center force position. The average of all 600 of these corrected residuals gives them equal weight.

The program then calculates and stores an array of volt/velocity ratios along with acquisition date and time. To eliminate polarity related offsets, consecutive values are combined in groups of three, weighted by 0.25/0.5/0.25 to correct for presumed linear drift of offsets in the DVMs, wire thermals, etc. The 18 “paired” values are subsequently averaged to provide a single volt/velocity ratio. Stored as text for further spreadsheet calculation of the final watt values are the time, individual ratio interpolated for the force position, standard deviation as the residuals from the curve fit, the “paired” ratio, and a flag indicating whether the program automatically excluded the curve from the average for statistical reasons. The combined average of the volt/velocity ratio is also stored. Typical noise values (residuals) per pass are about 1 × 10^−4^ to 5 × 10^−4^ at 481 V/(m/s), mostly from the residual noise of the 28 Hz vibration noise. Set average standard deviations range from 0.1 × 10^−6^ to 1 × 10^−6^. An estimate of the possible systematic Type B error involved in this profile fit technique is less than 0.02 µW/W. This improved scheme reduces the noise for individual velocity passes, yet the average has the same watt value as the simple 8th order fitting procedure.

### 5.2 Force/Current Ratio Analysis Programming

The post-experiment analysis is far simpler than the velocity mode. The data is just a voltage trace of the current through the 100 Ω resistor, recorded during a two minute period of balancing at the center position. The programmed routine performs a straightforward average, then adds in the reference voltage. The reference resistance is calculated and divided into the voltage to obtain the current. The force is calculated from the reference file, assuming constant values for the acceleration of gravity and the reference mass. The air buoyancy is calculated from the environmental variables and applied to each force point, assuming linear or higher order fitting, as mentioned earlier. Run times, current values, standard deviations, and force/current ratios, along with a total set average are stored in text format in the same file as the velocity data. The standard deviation for each average typically ranged from 0.05 × 10^−6^ to 0.1 × 10^−6^ of the ratio 481 N/A.

### 5.3 Watt Data

#### 5.3.1 Spreadsheet Analysis

After the post-experiment analysis program is run, final analysis is performed on a spreadsheet. A macro routine converts the analyzed data text file into a template format, which includes time plots of the analyzed ratios. All data plots are visually inspected to identify possible outliers, looking for unusual nonlinear drifts or clearly inconsistent points. Before elimination, outliers are crosschecked for large standard deviations within an analyzed data set. Some known intermittent external noise sources were RF interference (e.g., a nearby shortwave transmitter), mechanical vibrations (lawn mowers, local construction), or a coincidence with magnet supercurrent fluctuations (recorded separately). Occasionally a point stuck out, yet had a typical standard deviation. Unless visual inspection of the raw data could detect obvious noise spikes in the field profile or other problems, the point was retained. If a particular data point is to be eliminated, the three data points included in the offset combination are also deleted and an average value for each set of data is recalculated accordingly. A single watt data point is calculated by taking the ratio of the force/currrent average and a linear interpolation of the time bracketing volt/velocity averages. Finally, the correction for gravity tides is added to these watt values. The tides are calculated separately for fixed time intervals by the USGS software mentioned earlier. From this calculated list of tidal corrections, a watt correction is applied, coinciding to the nearest 7 minutes of the averaged time of the force/current average. Though the applied tide value might differ from an exactly timed value by as much as effective 6 nW/W, this error would occur randomly.

**Fig. 16:**
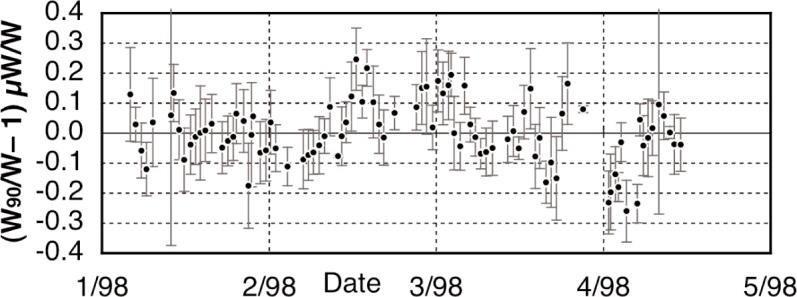
Daily averages of points used in the final calculation. Plot label refers to watt units (Eq. 10). Error bars are the Type A standard error for each day’s set. Day-to-day variations are small, though larger than expected for random statistical fluctuations. There is no long-term linear drift.

**Fig. 17:**
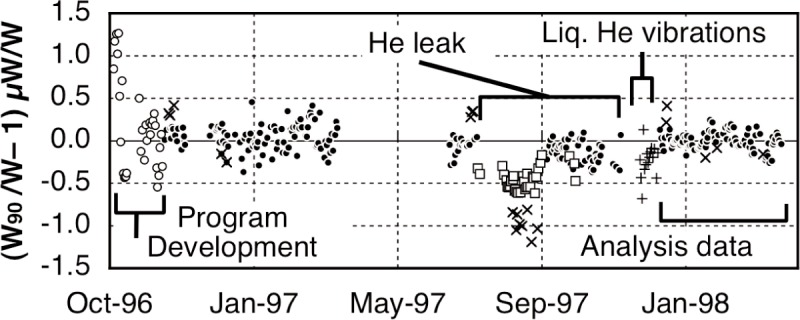
The record of daily averages of the watt values. Error bars are omitted for clarity. Open circles ○ are from early in program and procedure development. X points show the results of special tests outside of normal parameters. Squares □ are during known helium leaks. Plus + points have large velocity mode noise from helium vibrations. Solid points • have normal parameters and no obvious control problems.

The standard deviation (SD) of a day’s watt values provides a quick gauge of the relative quality of each day’s data. A good day’s run has a SD of about 0.15 µW/W. A typical run consists of about 12 points. Weekend and holiday runs could last for the full 2 days, although the time was usually broken up into smaller segments to allow for status checks or liquid helium transfers. Two databases are maintained: one of all the individually calculated watt points, on which the final calculations are performed, and another of the daily averages with the day’s SD and the SD of the mean. It is much easier to see the consistency of the watt values and the scatter from the plot of the daily averages (Figs. 16, 17). Since reference standards needed recalibration over the many months of data collection, each run was reanalyzed as necessary to account for any changes in applied reference values. Some sets of data were analyzed using slightly different calculation routines, to check for calculation or programming biases. These tests typically altered individual values within a day’s run by less than 0.1 µW/W, while the daily average values changed by an order of magnitude less.

#### 5.3.2 Interpreting the Data

The data from the final 3 months also compare well with data taken over the previous 2 years (Fig. 17). The earlier data are not included in the final analysis for several reasons. Mainly, there were significant improvements made in the system during this time, especially in alignment procedures. There was the elimination of helium gas from the local environment, improved temperature measurement and control, and closer maintenance of reference standards. This is seen in the reduced scatter over time. The overall differences in the daily averages for runs under control do not exceed ±0.5 µW/W. The match between July 1997 data and the final data is especially good. At that time, most of the operating procedures were in place, the alignments were stable, and the runs were very quiet. A test had shown negligible helium in the downstairs room air. Only the last 2 days after Aug. 2, 1997 show a sudden shift in the result, which continued when operations were resumed in late August. We assume that a major helium gas leak began at this time. A broken rubber hose in the helium exhaust line that had a tendency to burst during transfers was found, and a transfer had occurred on Aug. 2. Over the next few months, the values were still shifted until more helium leaks were found and repaired, including a cracked O-ring seal at the top of the Dewar, which could not be repaired in place. Eventually, the entire volume about the Dewar was lined with aluminum foil and heavy plastic to contain most of the exhaust gas plumbing. A fan ran continuously to vent the air in this space outside of the building. A hood with a similar vent fan was placed over the Josephson array Dewar, and extra effort went into minimizing the exhaust of any helium gas into the room during liquid transfers. However, even then, there was not an immediate return to the previous value. During this time, the liquid helium transfer line became blocked, requiring a quick warm up of the Dewar. After cooling again, a large helium thermo-acoustic vibration began, causing about five times more noise on the volt/velocity traces. Until this vibration died out, the watt values were again offset. It was not clear why these vibrations caused an offset.

#### 5.3.3 Finalized Data Set

After 3.5 months of consistent runs, from Jan. 6 to Apr. 18, 1998, the experiment reached its scheduled shut down time. A total of 989 points had been accumulated, and the final analysis was conducted on this data set. In going over these data, we made a list of the few corrections that needed to be applied. Most notably, after the mass was calibrated, it showed evidence of a drift, so a linear drift correction to the mass was incorporated into the data on the spreadsheet. A few other instruments were also recalibrated, such as the pressure meters, and these corrections were also applied. We did not do a blind test with a sealed-calibration reference, but there was no particular attention paid to the continuously changing average, only to the trend of the daily averages. We did notice that the corrections caused the data to shift up and down, but never far outside the typical scatter that was being obtained as far back as 1996. Only when the corrections list was checked off did we look closely at the final resulting average of all the points.

**Fig. 18:**
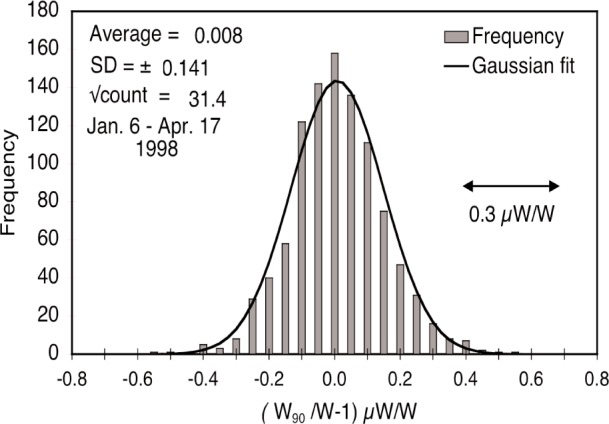
Histogram and Gaussian fit to the watt data incorporated into the final evaluation.

The average value (W_90_/W – 1), Eq. (10) of the full data set of 989 points is 0.008 µW/W. The standard deviation of this total set is 0.141 µW/W. The data fit a Gaussian distribution (Fig. 18). The standard error for this set would normally be about 10^−9^, but some periodicity is apparent in the daily average plots. Figure 16 shows the daily averages with standard error bars for each day’s set, usually 10–15 points. As a test for white noise or periodicity in the data, we grouped data into sections of increasing point count and looked to see when the group averages began to demonstrate $1/\sqrt{n}$ random scatter behavior. The behavior of the standard deviations changes at groupings of about 50 points, roughly 4 to 5 days of data. This also coincides with the typical interval of liquid helium transfers. Not only the changing weight of the liquid, but also the variation in thermal expansion of the solenoid support rods are known to affect the field profile. Close examination of the profile curves showed small but noticeable changes from day to day (Fig. 15 inset). The recalculation of the profile from each day’s run helped minimize the effect of these variations. But from the rapid room temperature changes during helium transfers, the alignment probably was also affected. It’s not clear whether this is the only cause. Given this nonrandom aspect of the short term data, we choose to calculate the standard error by using the square root of 19, representing the number of groups of 50 points in our data set. Thus the statistical standard error for the data set is calculated as 0.030 µW/W. We believe the larger, long term variations are likely due to the Type B sources of uncertainty. A root mean square (RMS) combination of the Type A with the RMS combination of the Type B uncertainties listed in Table 1, 0.082 µW/W, gives a relative combined uncertainty of 0.087 µW/W.

### 5.4 Conclusions

#### 5.4.1 Planck Constant Determination

**Fig. 19:**
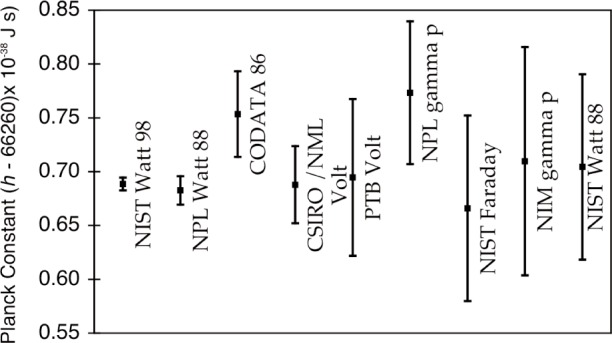
A comparison of the NIST 1998 evaluation with respect to other laboratory evaluations. NPL (England), CSIRO (Australia), PTB (Germany), NIM (Peoples Republic of China).

From the resulting watt value, and the theoretical calculations of Sec. 1, the Planck constant is determined as 6.626 068 91(58) × 10^−34^ J · s. The relative combined standard uncertainty is 8.7 × 10^−8^. A number of other fundamental physical constants are also determined by this result, as listed in Table 2. The last three values are included in the table, since they are used in the calculation of the other values. Figure 19 illustrates how this NIST evaluation fits in with respect to other laboratory evaluations of previous years, including the CODATA assignment of 1986.

#### 5.4.2 Epilogue—2004

Since the version of this experiment described here was concluded, a new version has been built. It incorporates improvements meant to eliminate the indirect references, mainly by enclosing the balance wheel and induction coil in a vacuum chamber. This chamber is in a larger room, with RF screening of the room and instrument areas. Certain mechanical and electronic inadequacies identified in the previous version are improved. Not much of the original system’s mechanical components remain in the new system, but parts have been saved and as studies are made on the new components, they are also made on the old ones. Some severe, systematic error sources have been found during construction and testing of the new system, but these have been solved or did not apply to the old system. No major additional systematic errors have yet been identified for parts of the old system.

Some noise sources are better understood. Chief among these is the vibration-related noise in the velocity data. It arises from flexing within the induction coil, which imparts phase error between the CM velocity relative to voltage due to imperfect placement of the interferometers. With a new induction coil (the third try), stiffened by a ceramic form and fitted with critically placed interferometer mirrors, velocity noise has been reduced by more than a factor of 10. Old and new knife edges have been separately studied. Even with better materials, the hysteretic effects, though far less, still remain. The coil support structures are modified to improve alignment check sensitivity, which greatly increased the effectiveness of alignment routine steps 4 and 5. Adjustments to the inductor coil angle and the mass pan position are also far easier. The stability of the alignments is improved both by these structural changes and also improved control programs on faster computers that eliminate sudden jerks or accidental bumps. A programmable Josephson array voltage system is now incorporated for direct voltage comparisons. By early 2004, these changes have combined to achieve typical within day watt value standard deviations of 0.02 µW/W. Compare this to the previous system, where a run’s standard deviation of 0.15 µW/W was a good day. Daily variations ranging over ±0.05 µW/W seem to indicate long period error sources yet to be discovered. The goal of this new version is to reduce the experimental uncertainty by a factor of 10 from 1998. After about 5 years of rebuilding and testing, this goal seems within our reach.

## Figures and Tables

**Table 1:** The uncertainty budget for the 1998 determination of Planck’s constant. Values are for *k* = 1

Standard uncertainty source	nW/W
Reference Units	
Voltage	30
Mass	20
Resistance	8
Length	5
Frequency	5
Gravity	7
External Effects	
Refractive index	43
Mass buoyancy	23
Alignments	40
Leakage resistance	20
Magnetic flux z-profile fit	20
Knife edge Hysteresis	20
EMI induced offsets	10

RSS sub total	82

Statistical type A	30

Combined standard uncertainty	87

**Table 2:** Evaluation of the Planck constant and other fundamental physical constants that are also determined. The last three constants are determined by other means, but are included since they are used in some of the other determinations

Constant	Symbol	Value	Unc. ur (10^−8^)
Planck constant	*h*	6.626 068 91(58) X10^−34^ J s	8.7 this work
Josephson constant (SI)	*K*_J_ = 2*e*/*h*	483 597.892(21) GHz/V	4.4 this work
Electron mass	*m*_e_	9.109 382 11(80) X10^−31^ kg	8.8 this work
Proton mass	*m*_p_	1.672 621 62(15) X10^−27^ kg	8.9 this work
Avogadro constant	*N*_A_	6.022 141 84(52) X10^23^ mol^−1^	8.7 this work
Elementary charge	*e*	1.602 176 48(7) X10^−19^ C	4.4 this work
Josephson constant	K_J−90_	483 597.9 GHz/V	exact (CIPM)
von Klitzing constant	*R*_K−90_	25 812.807 V	exact (CIPM)
*1*/(fine-structure constant)	*1*/*α*	137.035 999 93(52)	0.38 [21]
Rydberg constant	*R*_∞_	10 973 731.568 639(91) m^−1^	0.000 83 [22]
Electron’s atomic mass	*m*_e_/*m*_u_	0.000 548 579 911 1(12)	0.021 [23]
